# Parental COVID-19 vaccine hesitancy for children with neurodevelopmental disorders: a cross-sectional survey

**DOI:** 10.1186/s41182-022-00415-6

**Published:** 2022-03-21

**Authors:** Mohammad Ali, Tasnuva Shamarukh Proma, Zarin Tasnim, Md. Ariful Islam, Tania Akter Urmi, Sohel Ahmed, Abu-sufian Sarkar, Atia Sharmin Bonna, Umme Salma Khan

**Affiliations:** 1 Department of Physiotherapy and Rehabilitation, Uttara Adhunik Medical College and Hospital, Uttara Model Town, Dhaka, 1230 Bangladesh; 2 Hasna Hena Pain Physiotherapy and Public Health Research Center (HPRC), Uttara Model Town, Dhaka, 1230 Bangladesh; 3 Advanced Physiotherapy and Rehab Solution, Women’s Children’s and General Hospital, Dhanmondi, Dhaka, 1209 Bangladesh; 4 Department of Neurosurgery, Ibrahim Cardiac Hospital and Research Institute, Dhaka, Bangladesh; 5 Zaman Mordan Hospital, Sherpur Sadar, Sherpur, 2100 Bangladesh; 6 Jatio Protibondhi Seba O Sahajjo Kendro, Gopalgonj Sadar, Gopalgonj, 8100 Bangladesh; 7 Department of Physiotherapy, Mount Adora Hospital, Akhalia, Sylhet, 3100 Bangladesh; 8 Bashundhara Kings Football Club, Bashundhara R/A, Dhaka, 1229 Bangladesh; 9 Save the Children, Gulshan, 2, Dhaka, 1212 Bangladesh; 10 University of Turku, 20014 Turun yliopisto, Finland

**Keywords:** Bangladesh, COVID-19, Pediatrics, Neurodevelopmental disorders, Parental vaccine hesitancy

## Abstract

**Background:**

Little is known about parental coronavirus disease 2019 (COVID-19) vaccine hesitancy in children with neurodevelopmental disorders (NDD). This survey estimated the prevalence and predictive factors of vaccine hesitancy among parents of children with NDD.

**Methods:**

A nationally representative cross-sectional survey was conducted from October 10 to 31, 2021. A structured vaccine hesitancy questionnaire was used to collect data from parents aged ≥ 18 years with children with NDD. In addition, individual face-to-face interviews were conducted at randomly selected places throughout Bangladesh. Multiple logistic regression analysis was conducted to identify the predictors of vaccine hesitancy.

**Results:**

A total of 396 parents participated in the study. Of these, 169 (42.7%) parents were hesitant to vaccinate their children. Higher odds of vaccine hesitancy were found among parents who lived in the northern zone (AOR = 17.15, 95% CI = 5.86–50.09; *p* < 0.001), those who thought vaccines would not be safe and effective for Bangladeshi children (AOR = 3.22, 95% CI = 1.68–15.19; *p* < 0.001), those who were either not vaccinated or did not receive the COVID-19 vaccine themselves (AOR = 12.14, 95% CI = 8.48–17.36; *p* < 0.001), those who said that they or their family members had not tested positive for COVID-19 (AOR = 2.13, 95% CI = 1.07–4.25), and those who did not lose a family member to COVID-19 (AOR = 2.12, 95% CI = 1.03–4.61; *p* = 0.040). Furthermore, parents who were not likely to believe that their children or a family member could be infected with COVID-19 the following year (AOR = 4.99, 95% CI = 1.81–13.77; *p* < 0.001) and who were not concerned at all about their children or a family member being infected the following year (AOR = 2.34, 95% CI = 1.65–8.37; *p* = 0.043) had significantly higher odds of COVID-19 vaccine hesitancy.

**Conclusions:**

Given the high prevalence of vaccine hesitancy, policymakers, public health practitioners, and pediatricians can implement and support strategies to ensure that children with NDD and their caregivers and family members receive the COVID-19 vaccine to fight pandemic induced hazards.

## Background

Vaccine hesitancy has been declared as one of the top 10 global health threats by the World Health Organization in 2019 [1]. Vaccine hesitancy can be defined as a delay in refusal or acceptance of a vaccine during the availability of a vaccination service [2]. For children, parental vaccine hesitancy is a significant barrier to a smooth vaccination program for fighting vaccine-preventable diseases [3]. In addition, previous studies have suggested that parents of children with a neurodevelopmental disorder (NDD) have a significantly higher vaccine hesitancy than parents with neurotypical children [4, 5].

Many parents of a child with NDD believe that vaccines can negatively impact their child's disability conditions [6]. Research has further demonstrated that parents of a child with NDD are also vaccine-hesitant for their other healthy children [7]. Conversely, children with NDD and their families are significant victims of the current coronavirus disease 2019 (COVID-19) pandemic [8]. Despite the limitations of public health data, some evidence suggests that children with NDD might be disproportionately affected by the COVID-19 itself or the pandemic’s impact on receiving services. In addition, children with NDD often have medical conditions that contribute to a higher risk of severe COVID-19 disease [9].

Furthermore, NDD patients affected by COVID-19 can experience difficulties accessing required healthcare and possess other characteristics that increase their difficulties faced due to COVID-19. These include mobility limitation, direct supervision requirements, challenges practicing prophylactic measures, and communicating disease symptoms [9]. Furthermore, evidence suggests that COVID-19 incidence, hospitalization, ICU admission, and death are much higher among COVID-19 patients with NDD [10].

Previous studies have found a high prevalence of COVID-19 vaccine hesitancy among parents for their neurotypical children in several settings; this was a matter of great concern when discussing immunization of an optimum population percentage to gain community herd immunity for fighting the pandemic [11, 12]. In addition, existing literature suggested that the higher COVID-19 vaccine hesitancy among parents of a child with NDD would raise further concern worldwide [13].

In Bangladesh, the COVID-19 vaccine roll-out was initiated on January 27, 2021, for adults aged 18 years and above [14]. Vaccination among students aged 12–17 started on November 1, 2021 [15]. However, very little is known about the COVID-19 vaccine hesitancy among the parents of children aged under 18. Furthermore, it is crucial to determine whether the higher prevalence of vaccine hesitancy among parents of a child with NDD is consistent with the COVID-19 vaccination program, considering the public health importance of widespread COVID-19 vaccination in Bangladesh and other parts of the world. In addition, it is vital to identify other factors associated with vaccine hesitancy in this population that could inform the design of targeted, preemptive vaccine interventions. With this in mind, our objectives were to (1) estimate the prevalence of vaccine hesitancy within a nationally representative sample of parents of children with NDD and (2) identify the predictors of vaccine hesitancy considering socioeconomic and behavioral factors and COVID-19 threat perception.

## Methods

### Study design and participants

Anonymous data were collected from October 10 to 31, 2021, for this nationally representative cross-sectional survey. Individuals aged ≥ 18 years who were parents of at least one child aged under 18 years with neurodevelopmental disorders were eligible for this study. Individualized interviews using a structured questionnaire were conducted in homes and health care centers. Parents aged < 18 years were excluded from the study. A margin of 5% error, a confidence level of 95%, and a response distribution of 50% were used to calculate the sample size to target fathers/mothers of approximately 8 million children and secure a minimum sample size of 384 participants [16, 17]. Data were collected from 408 individuals.

### Sampling technique

Data were collected from all eight divisions in Bangladesh. We identified approximately 150 government and non-government centers dealing with children with NDD. In addition, we visited 38 randomly selected centers to collect data of the parents. Approximately 450 parents were conveniently approached for the interviews.

### The questionnaire

Previously employed vaccine hesitancy and COVID-19 threat perception questionnaire modified for parents were used in this study [14]. In the first part, parents were asked about the likelihood of vaccinating their children with NDD. Vaccine hesitancy was measured using the question, “If a vaccine that would be effective against coronavirus among children was available, how likely are you to get your children with NDD vaccinated?” The response options for this question were “very likely,” “somewhat likely,” “not likely,” and “definitely not.” Participants were also asked two questions regarding the perceived COVID-19 threat: (1) “How likely is it that your children or a family member could get infected with coronavirus in the next one year?” The response options for this were “very likely,” “somewhat likely,” “not likely,” and “definitely not.” (2) “How concerned are you that your children or a family member could get infected with COVID-19 in the next year?” Here, response options were “very concerned,” “concerned,” “slightly concerned,” and “not concerned at all.” The second part of the questionnaire included a wide range of sociodemographic, behavioral, and COVID-19-related questions regarding both the child and parent.

### Data analysis

Descriptive statistics were employed to elaborate on the demographic characteristics of the study participants. *Χ*^2^ test or Fisher’s exact test was used to calculate vaccine hesitancy proportions and draw comparisons between subgroups. Responses were compared for various sociodemographic characteristics by dichotomizing the variable as positive (“very likely” and “somewhat likely”) or a negative (“not likely” and “definitely not”) attitude toward the COVID-19 vaccine, indicating the prevalence of vaccine hesitancy. Multiple logistic regression analyses were performed with vaccine hesitancy as a dependent variable and sociodemographic characteristics and perceived COVID-19 threat as predictor variables for vaccine hesitancy to calculate adjusted odds ratios (AOR) with a 95% confidence interval (CI). In this model, we included factors significantly associated with vaccine hesitancy in the descriptive analysis. The Hosmer–Lemeshow goodness-of-fit test was employed to ensure that the models adequately fit the data. Statistical significance was set at *p* < 0.05. SPSS version 0.22.0 (IBM Corp., USA) was used for all data analyses.

## Results

With a 90.7% response rate, 408 parents provided informed consent and data for this study. Seven parents refused to answer the entire question and were thus excluded. We also excluded five more data points for inconsistent answers to the questions. Finally, a total of 396 parents (60.4% men) aged 34.54 ± 6.56 years (mean ± standard deviation) included in the analysis. Of the children, 60.9% were male, and 40.2% were in the 0–4-year-old group. The highest number of parents (27.5%) were in the 31–35-year-old group. Overall, 87.4% of parents were Muslim, 70.7% were belonged to nuclear family members, 44.2% had two children, 29.8% had a low education level, 24.2% were service holders, and 33.1% had a high household income. Among all participants, 49.2% were from the village, 62.6% lived in the central zone, including Dhaka, 76.3% were not tobacco users, 76.8% were regular religious practitioners, and 37.9% were politically neutral respondents. However, 3.3% of parents did not adhere to the standard government vaccination (other than COVID-19) programs, and 53.5% remained skeptical about COVID-19 vaccine safety and effectiveness for Bangladeshi children. Furthermore, 20.5% of parents were either not vaccinated or did not receive the COVID-19 vaccine themselves, but 39.6% of parents reported that they or their family members had tested positive for COVID-19 earlier, and 8.6% had lost a loved one due to COVID-19. Details of the responses to the questions regarding the likelihood of children or family members’ COVID-19 infection and the level of concern about children or family members being infected in the next year are shown in Table 1.

**Table 1:** Descriptive analysis: sociodemographic characteristics, COVID-19 threat, and parental vaccine hesitancy

Variables	Total sample *n* (%)	Likelihood of vaccinating children		*p* value
		Not likely/definitely not *n* (%)	Very likely/somewhat-likely *n* (%)	
All participants	396 (100)	169 (42.7)	227 (57.3)	N/A
Children’s age group (in year)				
0–4	159 (40.2)	92 (57.9)	67 (42.1)	**< 0.001**
5–9	156(39.4)	60 (38.5)	96 (61.5)	
10–14	63 (15.9)	13 (20.6)	50 (79.4)	
15–17	18 (4.5)	4 (22.2)	14 (77.8)	
Children’s gender				
Male	241 (60.9)	104 (43.2)	137 (56.8)	0.811
Female	155 (39.1)	65 (41.9)	90 (58.1)	
Parents’ age group (in year)				
18–25	27 (6.8)	13 (48.1)	14 (51.9)	**< 0.001**
26–30	94 (23.7)	49 (52.1)	45 (47.9)	
31–35	109 (27.5)	57 (52.3)	52 (47.7)	
36–40	101 (25.5)	36 (35.6)	65 (64.4)	
41–45	45 (11.4)	14 (31.1)	31 (68.9)	
46–50	15 (3.8)	0 (0)	15 (100)	
≥ 51	5 (1.3)	0 (0)	5 (100)	
Parents’ gender				
Female	157 (39.6)	74 (47.1)	83 (52.9)	0.146
Male	239 (60.4)	95 (39.7)	144 (60.3)	
Marital status				
Married	376 (94.9)	163 (43.4)	213 (56.6)	0.239
Divorced or widow	20 (5.1)	6 (30)	14 (70)	
Religion				
Muslim	346 (87.4)	149 (43.1)	197 (56.9)	0.521
Hindu	47 (11.9)	20 (42.6)	27 (57.4)	
Buddha	1 (.3)	0 (0)	1 (100)	
Cristian	2 (.5)	0 (0)	2 (100)	
Type of family				
Joint family	116 (29.3)	56 (48.3)	60 (51.7)	0.147
Nuclear family	280 (70.7)	113 (40.4)	167 (59.6)	
Number of children				
One	131 (33.1)	66 (50.4)	65 (49.6)	0.090
Two	175 (44.2)	67 (38.3)	108 (61.7)	
Three or more	90 (22.7)	36 (40)	54 (60)	
Educational qualification				
≤ High school	118 (29.8)	46 (39)	72 (61)	0.176
College education	99 (25)	44 (44.4)	55 (55.6)	
Graduate	103 (26)	52 (50.5)	51 (49.5)	
Post graduate	76 (19.2)	27 (35.5)	49 (64.5)	
Occupation				
Service	96 (24.2)	41 (42.7)	55 (57.3)	0.419
Business	60 (15.2)	32 (53.3)	28 (46.7)	
Unemployed	21 (5.3)	10 (47.6)	11 (52.4)	
Student	6 (1.5)	2 (33.3)	4 (66.7)	
Home maker	157 (39.6)	65 (41.4)	92 (58.6)	
Healthcare	26 (6.6)	7 (26.9)	19 (73.1)	
Daily labor	30 (7.6)	12 (40)	18 (60)	
Monthly household income (৳)				
< ৳15 000	103 (26)	45 (43.7)	58 (56.3)	0.973
৳ 15,000–30,000	86 (21.7)	35 (40.7)	51 (59.3)	
৳ 31,000–45,000	76 (19.2)	32 (42.1)	44 (57.9)	
> ৳ 45,000	131 (33.1)	57 (43.5)	74 (56.5)	
Current residence type				
Own	186 (47)	81 (43.5)	105 (56.5)	0.907
Rented	190 (48)	79 (41.6)	111 (58.4)	
Others	20 (5)	9 (45)	11 (55)	
Permanent address				
Village	195 (49.2)	88 (45.1)	107 (54.9)	0.128
Semi city	84 (21.2)	40 (47.6)	44 (52.4)	
City	117 (29.5)	41 (35)	76 (65)	
Current living location				
Central zone	248 (62.6)	107 (43.1)	141 (56.9)	**< 0.001**
North zone	74 (18.7)	44 (59.5)	30 (40.5)	
South zone	74 (18.7)	18 (24.3)	56 (75.7)	
Present tobacco user				
No	302 (76.3)	127 (42.1)	175 (57.9)	0.653
Yes	94 (23.7)	42 (44.7)	52 (55.3)	
Regular religious practice				
No	92 (23.2)	39 (42.4)	53 (57.6)	0.950
Yes	304 (76.8)	130 (42.8)	174 (57.2)	
Political affiliation				
Ruling party	126 (31.8)	45 (35.7)	81 (64.3)	0.160
Opposition	49 (12.4)	19 (38.8)	30 (61.2)	
Neutral	150 (37.9)	73 (48.7)	77 (51.3)	
Prefer not to say	71 (17.9)	32 (45.1)	39 (54.9)	
Vaccinated/plan to vaccinate children under regular govt. vaccination programs (other than COVID-19)				
No	13 (3.3)	6 (46.2)	7 (53.8)	0.779
Yes	383 (96.7)	163 (42.6)	220 (57.4)	
Do you think the COVID-19 vaccine will be safe and effective for Bangladeshi children?				
No	22 (5.6)	19 (86.4)	3 (13.6)	**< 0.001**
Yes	162 (40.9)	12 (7.4)	150 (92.6)	
Skeptical	212 (53.5)	138 (65.1)	74 (34.9)	
Have you taken or planned to take the COVID-19 vaccine?				
No	81 (20.5)	64 (79)	17 (21)	**< 0.001**
Yes	315 (79.5)	105 (33.3)	210 (66.7)	
Were you or your family member(s) tested positive for COVID-19?				
No	239 (60.4)	121 (50.6)	118 (49.4)	**< 0.001**
Yes	157 (39.6)	48 (30.6)	109 (69.4)	
Have you lost any of your family member(s) for COVID-19?				
No	362 (91.4)	163 (45)	199 (55)	**0.002**
Yes	34 (8.6)	6 (17.6)	28 (82.4)	
Perceived likelihood of children or family members’ infection in the next year				
Very likely	56 (14.1)	9 (16.1)	47 (83.9)	**< 0.001**
Somewhat likely	240 (60.6)	102 (42.5)	138 (57.5)	
Not likely	73 (18.4)	40 (54.8)	33 (45.2)	
Definitely not	27 (6.8)	18 (66.7)	9 (33.3)	
Level of concern about children or family members’ infection in the next year				
Very concerned	49 (12.4)	14 (28.6)	35 (71.4)	**< 0.001**
Concerned	128 (32.3)	38 (29.7)	90 (70.3)	
Slightly concerned	122 (30.8)	56 (45.9)	66 (54.1)	
Not concerned at all	97 (24.5)	61 (62.9)	36 (37.1)	

Bold faces are significant at a 5% significance level

**Fig. 1:**
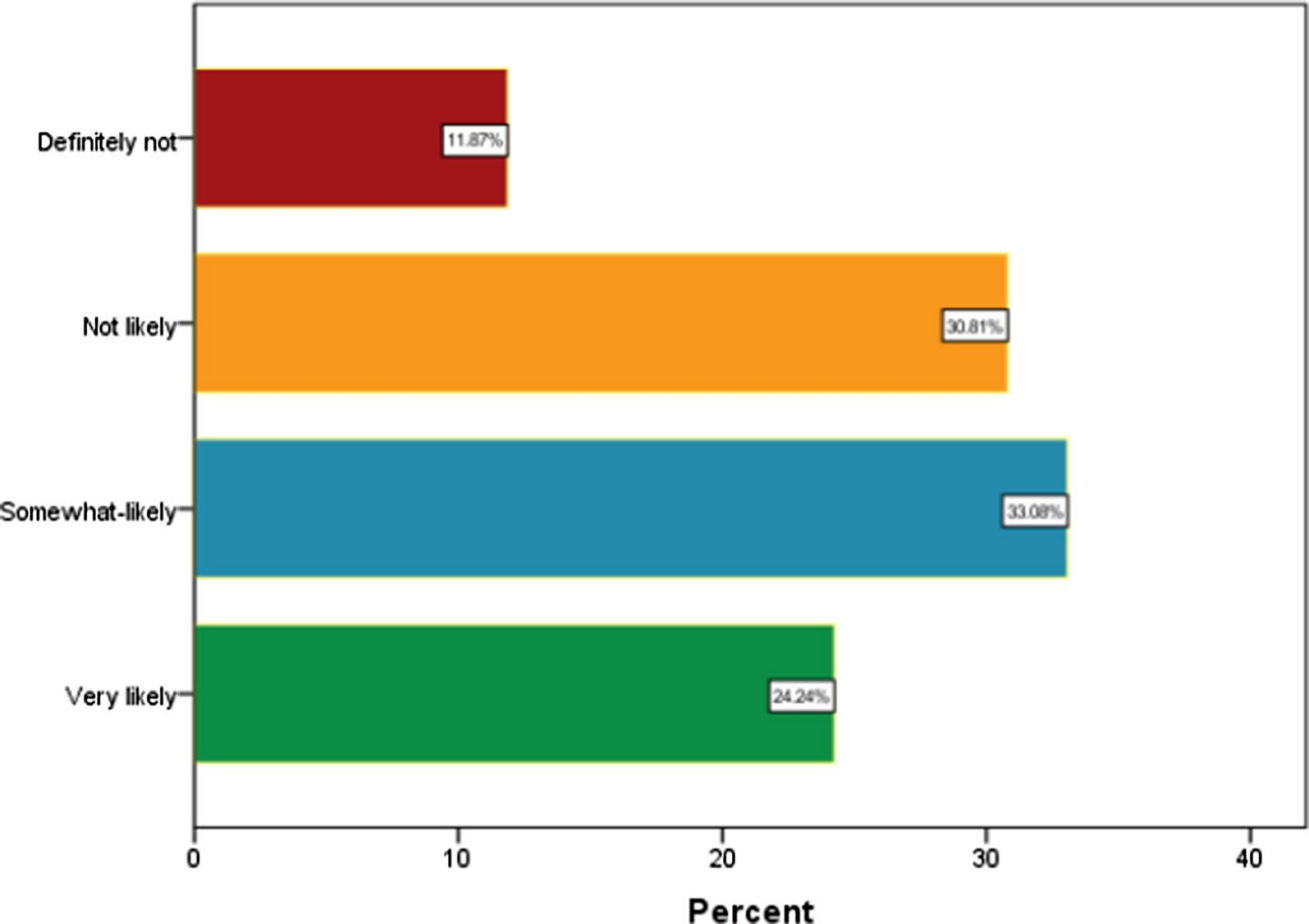
Likelihood of COVID-19 vaccine acceptance/refusal by Bangladeshi parents for children with neurodevelopmental disorders

Overall, 42.7% (CI = 37.82–47.57) of parents were hesitant to vaccinate their children with NDD against COVID-19. Table 1 displays the result of descriptive analysis. The highest prevalence of vaccine hesitancy was observed among parents of children aged 0–4 years (57.9%; *p* < 0.001), parents aged 31–35 years (52.3%; *p* < 0.001), those living in the northern zone (59.5%; *p* < 0.001), those who did not believe the vaccine will be safe and effective for Bangladeshi children (86.4%; *p* < 0.001), those who were either not vaccinated or chose not receive the COVID-19 vaccination themselves (79%, *p* < 0.001). Furthermore, vaccine hesitancy was highest among parents who said that they or their family members had not tested positive for COVID-19 (50.6%; *p* < 0.001) and those who did not lose any family member due to COVID-19 (45%; *p* = 002). Moreover, participants who were not likely to believe that their children or a family member could be infected with COVID-19 the following year (66.7%; *p* < 0.001) and those who were not concerned at all about their children or a family member getting infected in the following year (62.9%; *p* < 0.001) showed high levels of COVID-19 vaccine hesitancy. Figure 1 shows the breakdown of vaccine acceptance or refusal. Table 2 displays the results of multiple logistic regression analyses. The subgroup of participants with the highest odds of vaccine hesitancy were those who lived in the northern zone (AOR = 17.15, 95% CI = 5.86–50.09; *p* < 0.001), who thought vaccines would not be safe and effective for Bangladeshi children (AOR = 3.22, 95% CI = 1.68–15.19; *p* < 0.001), who were either not vaccinated or did not receive the COVID-19 vaccine themselves (AOR = 12.14, 95% CI = 8.48–17.36; *p* < 0.001), who said that they or their family members had not tested positive for COVID-19 (AOR = 2.13, 95% CI = 1.07–4.25), and who did not lose a family member to COVID-19 (AOR = 2.19, 95% CI = 1.07–4.61; *p* = 0.040). Furthermore, parents who were not likely to believe that their children or a family member could be infected with COVID-19 the following year (AOR = 4.99, 95% CI = 1.81–13.77; *p* < 0.001) and not concerned at all about their children or a family member getting infected the next year (AOR = 2.34, 95% CI = 1.65–8.37; *p* = 0.043) had significantly higher odds of COVID-19 vaccine hesitancy.

**Table 2:** Multiple logistic regression: predictors of parental vaccine hesitancy in study participants

Variables	Adjusted OR	SE	95% CI		*p* value
Children’s age group (in year)					
0–4	1.57	0.83	0.31	8.02	0.588
5–10	0.61	0.82	0.12	3.04	0.550
11–14	0.41	0.86	0.08	2.23	0.304
15–< 18	References				
Parents’ age group (in year)					
18–25	Reference				
26–30	1.25	0.62	0.37	4.18	0.722
31–35	1.31	0.62	0.39	4.43	0.667
36–40	0.97	0.64	0.28	3.40	0.959
41–45	0.79	0.74	0.19	3.33	0.745
≥ 46	1.10	0.81	0.31	3.54	0.878
Current living location					
Central zone including Dhaka	7.25	0.45	2.98	17.67	**< 0.001**
North zone	17.15	0.55	5.87	50.09	**< 0.001**
South zone	Reference				
Do you think the COVID-19 vaccine will be safe and effective for Bangladeshi children					
No	3.22	0.79	1.68	15.19	**0.049**
Yes	0.04	0.39	0.02	0.09	**< 0.001**
Skeptical	Reference				
Have you taken or plan to take the COVID-19 vaccine					
No	12.14	0.18	8.48	17.36	** < 0.001**
Yes	Reference				
Were you or your family member(s) tested positive for COVID-19					
No	2.13	0.35	1.07	4.25	**0.032**
Yes	Reference				
Have you lost any of your family member(s) for COVID-19					
No	2.19	0.38	1.04	4.62	**0.040**
Yes	Reference				
Perceived likelihood of children or family members’ infection in the next year					
Very likely	Reference				
Somewhat likely	4.54	0.52	0.99	22.36	0.051
Not likely	4.71	0.63	1.32	15.67	**0.017**
Definitely not	4.99	0.79	1.81	13.78	**0.002**
Level of concern about children or family members’ infection in the next year					
Very concerned	Reference				
Concerned	0.69	0.58	0.22	2.16	0.527
Slightly concerned	1.04	0.56	0.34	3.21	0.943
Not concerned at all	2.34	0.65	1.65	8.37	**0.043**

Bold faces are significant at a 5% significance level

## Discussion

To the best of our knowledge, this is the first study to analyze parental COVID-19 vaccine hesitancy in parents of children with NDD. This nationally representative survey found a significantly high prevalence of parental vaccine hesitancy in Bangladesh. Logistic regression analysis suggested that location of residence, perception about vaccine safety and effectiveness for children, experience of family members being testing positive for COVID-19, and a family members’ death due to COVID-19 strongly predicted vaccine hesitancy. Furthermore, COVID-19 threat perceptions were significantly associated with parental vaccine hesitancy for their children with NDD.

COVID-19 vaccine hesitancy is generally high among Bangladeshi adults [14]; however, we found a higher parental vaccine hesitancy among parents for their children with NDD in this study (32.5% vs. 42.7%). This prevalence was also much higher than that of common non-COVID-19 vaccine hesitancy among parents of children with NDD in another country [4]. Nonetheless, a contemporary study conducted in Taiwan among caregivers of children with attention–deficit/hyperactivity disorder found that 37% of caregivers hesitated or refused to vaccinate their children against COVID-19 [18]. In addition, 42% and 52% of parents of healthy children in the USA and China, respectively, were hesitant to vaccinate their children against COVID-19 [12, 19].

In line with the findings of previous studies [19, 20], we found higher vaccine hesitancy among younger parents as well as parents of younger children. The safety and efficacy of the COVID-19 vaccine in children aged < 12 years remains unclear, which might cause higher vaccine hesitancy among parents of young children [21]. However, more studies are warranted to understand what drives young parents not to vaccinate their children against COVID-19. Conversely, in agreement with our previous study on the adult population, we found remarkably higher vaccine hesitancy among parents who lived in the central and northern zones of Bangladesh [14]. However, it is impossible to infer the cause of higher vaccine hesitancy in these areas from our data, thus indicating the need for additional studies to understand causal relationships.

Our analyses found that one-fifth of the participants were either not vaccinated or chose not receive the COVID-19 vaccination themselves. Unsurprisingly, 80% of the non-vaccinated parents were hesitant to vaccinate their children. Previous studies have also observed a significant association between parental willingness to get vaccinated and reported intentions to vaccinate children [19, 21]. However, we found a lower prevalence of parental vaccine hesitancy for their children among participants who reported that their family members tested positive for or died of COVID-19. Therefore, it can be assumed that perception of disease threats drove these parents to make a favorable decision regarding vaccination.

Disease threat perception is the key to theories of many health behaviors. A systematic review and meta-analysis concluded that the likelihood of risk, susceptibility, and severity from the disease is significantly associated with vaccine hesitancy [22]. Furthermore, a recent study revealed that high COVID-19 risk perception was associated with reduced vaccine hesitancy [23]. Another study suggested that reduced risk perception is associated with reduced COVID-19 vaccine uptake [24]. In agreement with these findings, our study found that perceived COVID-19 threat was one of the strongest predictors of parental vaccine hesitancy. In contrast, an empirical investigation revealed that, among others, the safety of the COVID-19 vaccine outweighs disease risk perception when predicting vaccine hesitancy [25]. Our study also found a high prevalence of vaccine hesitancy among parents who remained skeptical or did not perceive that the vaccine would be safe and effective for Bangladeshi children.

This study had several limitations. First, the cross-sectional nature of this study only portrays the community response at the time of the study. However, studies have found that vaccine hesitancy is complex in disposition and is adherence-specific, varying over time, location, and the perceived behavioral nature of the community [26–28]. Second, the inclusion of the Bangladeshi tribal population may help increase the generalizability of the findings. Despite these limitations, this is the first study to provide baseline evidence regarding COVID-19 vaccine hesitancy among parents of children with NDD, identifying a range of subgroups of the parents that must be considered during widespread vaccination discussions in low- and middle-income countries. Finally, face-to-face data collection from randomly selected regions throughout Bangladesh would have reduced nonresponse bias and provided a better representation of the population in the sample, thus increasing the generalizability of the study.

## Conclusions

Given the increased prevalence of underlying health conditions, suboptimal vaccination rates, and systemic inequities, children with NDD are likely to have a higher risk of being infected with COVID-19 and its outcomes. Policymakers, public health practitioners, and pediatricians should address the findings of this study when discussing and implementing strategies to ensure that children with NDD, their caregivers and family members, and service providers receive the COVID-19 vaccine. In addition, highlighting the unique considerations for COVID-19 vaccination for children with NDD can support equitable vaccination access for these children and their families.

## Data Availability

Data will be made available on request.

## Abbreviations

NDD: Neurodevelopmental disorders
AOR: Adjusted odds ratio
CI: Confidence interval
COVID-19: Coronavirus disease of 2019
